# How the brain regulates alcohol intake

**DOI:** 10.7554/eLife.82453

**Published:** 2022-09-14

**Authors:** Leigh C Walker, Paulo Pinares-Garcia, Andrew J Lawrence

**Affiliations:** 1 Florey Institute of Neuroscience and Mental Health, University of Melbourne Parkville Australia

**Keywords:** alcohol, striatum, insula, synaptic plasticity, Mouse

## Abstract

A neural pathway involved in goal-oriented behaviours becomes dysregulated during binge drinking and alcohol use disorder.

**Related research article** Haggerty DL, Muñoz B, Pennington T, Di Prisco GV, Grecco GG, Atwood BK. 2022. The role of anterior insular cortex inputs to dorsolateral striatum in binge alcohol drinking. *eLife* **11**:e77411. doi: 10.7554/eLife.77411.

Many people drink alcohol moderately at celebrations, work events and other social situations. However, patterns of excessive alcohol consumption within short periods, termed binge drinking, are becoming increasingly common and often lead to alcohol use disorder (sometimes referred to as alcoholism; Addolorato et al., 2018). Despite this, surprisingly little is known about the changes in the brain that drive binge drinking, and the neural pathways that govern this behaviour.

One theory is that alcohol use disorder develops when there is a shift in the brain regions that control behaviours associated with drinking alcohol. In moderate drinkers, alcohol consumption is regulated by a region of the brain called the dorsomedial striatum (DMS), which controls goal-oriented behaviours. In people with alcohol use disorder, this control shifts to the dorsolateral (DLS) striatum, a region that regulates habitual actions (Corbit et al., 2012).

Many brain regions send information to the DMS and the DLS. One of these regions is the anterior insula cortex (AIC), a part of the brain that is thought to integrate how our body is feeling inside with changes in the environment. This allows the AIC to drive motivated behaviours – this is, behaviours with a specific goal – including alcohol consumption (Jaramillo et al., 2018). Recent studies have shown that a neural pathway that travels from the AIC to the DLS is particularly vulnerable to alcohol exposure, especially when compared to the pathways that connect the AIC and the DMS, or to the projections the DLS receives from other brain regions (Muñoz et al., 2018).

Now, in eLife, Brady Atwood and colleagues at Indiana University School of Medicine – including David Haggerty as first author – have combined a mouse binge drinking model with elegant optogenetic and electrophysiology approaches to examine how alcohol consumption dysregulates the neural pathway that transmits inputs from the AIC into the DLS (Haggerty et al., 2022; see Figure 1).

**Figure 1.**
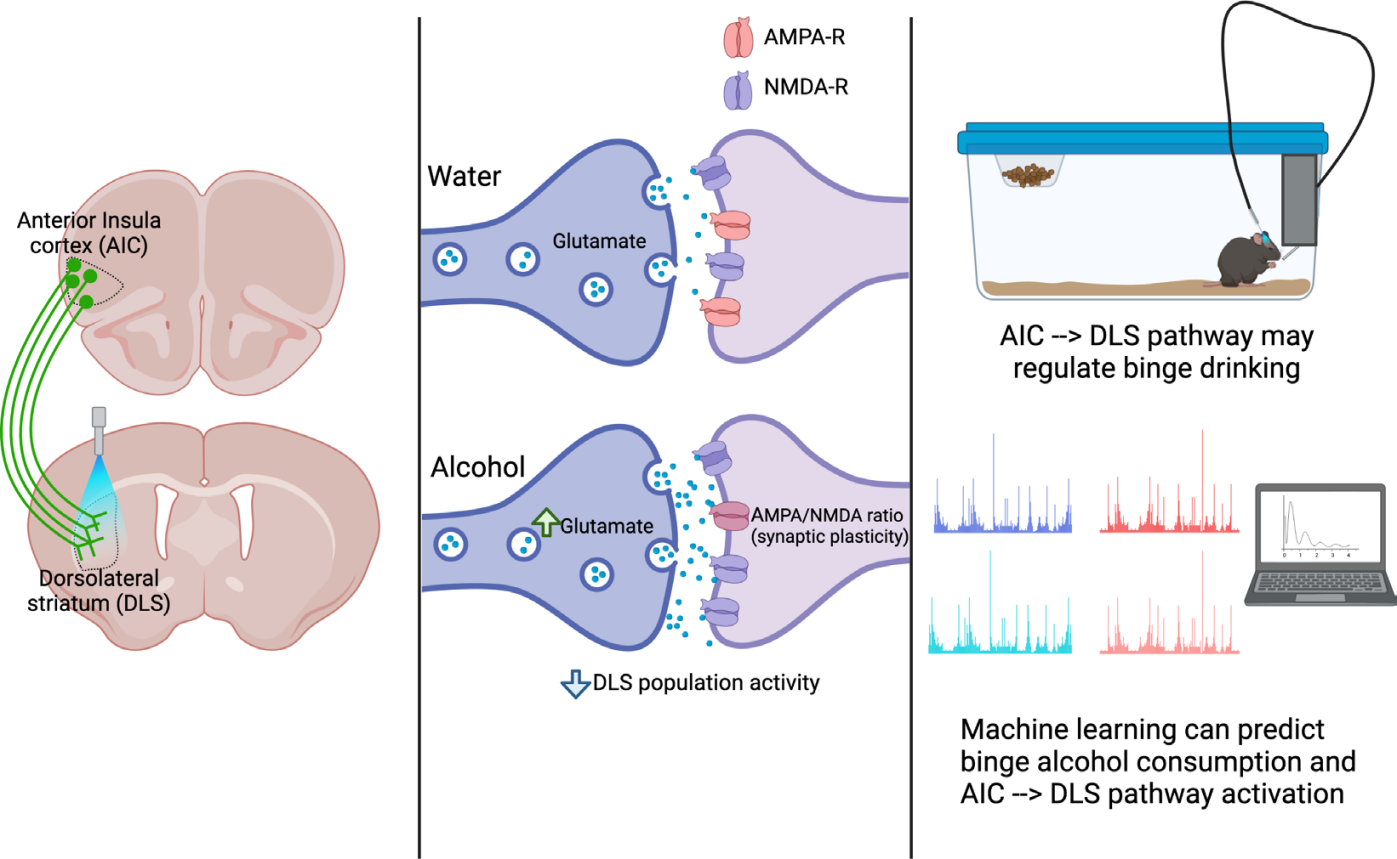
A pathway between the anterior insula cortex (AIC) and dorsolateral striatum (DLS) regulates alcohol binge drinking. Left: The projections of neurons from the AIC (green) into the DLS can be activated with light (a technique called optogenetics) to release glutamate into the DLS. Centre: The AIC to DLS pathway shows changes in its response to binge drinking alcohol (bottom), but not water (top). These changes include increased glutamate release at the synapse between AIC neurons (dark purple) and neurons in the DLS (light purple); synaptic plasticity (a change in the ratio between two glutamate receptors: AMPA-R and NMDA-R); and an overall decrease in the activity of DLS neurons. Right: activation of the pathway from the AIC to the DLS reduces alcohol consumption, indicating that this pathway can regulate binge drinking. Both the activation of the AIC to DLS pathway and binge alcohol consumption can be predicted using machine learning to analyse the dynamics of drinking behaviours, indicating a relationship between the pathway and alcohol intake.

Image made using BioRender.com.

First, Haggerty et al. assessed how neurons in the DLS responded to inputs from the AIC after three weeks of binge drinking alcohol. To perform this experiment, mice first underwent several binge drinking sessions over three weeks. These sessions lasted for zero (in the controls), two or four hours, during which the mice had access to either alcohol or water. After the three weeks, Haggerty et al. used light to control when neurons in the AIC sent signals into the DLS of these mice (a method termed optogenetics). This allowed Haggerty et al. to confirm their previous finding that stimulating neurons in the AIC of mice that have been binge drinking increases the release of glutamate (an excitatory neurotransmitter) into the DLS, compared to mice that only drank water (Muñoz et al., 2018).

Next, Haggerty et al. used the same mice to record the electrical activity of medium spiny neurons in the DLS to determine how they responded to signals from the AIC. In male mice, alcohol altered the electrical activity, making the network formed by these DLS neurons less responsive to inputs from the AIC, potentially supporting binge drinking behaviours. Notably, a single binge drinking session did not reproduce the same effects in the spiny neurons of the DLS as three weeks of binging, but the increase in glutamate release from the neurons in the AIC was maintained. Furthermore, none of these effects were seen in female mice.

To explore the role that the pathway between the AIC and the DLS plays in binge drinking, Haggerty et al. used optogenetics in male mice. This approach allowed the researchers to control the activity of glutamate-releasing neurons from the AIC that project into the DLS during a binge drinking session. The experiment showed that activation of this pathway, which was triggered by the mice licking for an alcohol reward, reduced the amount of alcohol the mice consumed, but not how much water they drank. Assessing the behavioural patterns of alcohol consumption within the session revealed that the mice that had received optogenetic activation consumed less alcohol at the beginning of the session. A parallel experiment showed that for these reductions in intake to occur, the mice needed to have consumed alcohol previously.

Haggerty et al. then used machine learning to analyse the dynamics of drinking behaviours, and built a predictive model that was able to reliably tell whether the mice had been drinking water or alcohol, and whether they had received optogenetic stimulation during the drinking session. The fact that this predictive model could be built highlights the relationship between the brain pathway connecting the AIC to the DLS and alcohol consumption. Finally, Haggerty et al. showed that the pathway did not alter other factors that influence alcohol consumption, such as sedation, increased perception of reward or anxiety levels.

Together these data delineate a pathway in the brain that is influenced by alcohol consumption, and may regulate ongoing alcohol intake, as well as the transition from binge drinking to alcohol use disorder. However, several unanswered questions remain: for example, what is the local mechanism within the DLS driving this response? Neurons in the striatum are highly regulated by neighbouring inhibitory cells, whose properties can be altered by alcohol (Patton et al., 2021). Whether the inputs from the AIC to the DLS directly activate inhibitory cells that govern local and/or network activity, or whether this occurs directly though medium spiny neurons remains to be explored.

Another important question is whether the changes observed by Haggerty et al. are due to alcohol itself or to the formation of a reward-related routine (habit). The DLS is critical to forming habits connected to drug and non-drug rewards (Yin et al., 2004). Therefore, while changes in brain plasticity and behaviour were only present after repeated binge drinking sessions, without comparing to another equal non-drug reward, such as sugar, the AIC to DLS pathway’s role in binge drinking remains equivocal.

Finally, another point for future consideration are the sex-specific effects of alcohol consumption that were observed only in the brains of male mice. Female mice in the experiments consumed more alcohol than their male counterparts, while in humans, more women are engaging in risky binge drinking behaviours (Grant et al., 2017). Understanding the underlying sex differences in the way that alcohol changes the brain, and the neurobiology that drives differences in binge drinking between the sexes may help provide better treatment options for alcohol use disorder in the future.
